# Supplementary material for: RUNX1 marks a luminal castration-resistant lineage established at the onset of prostate development
Source: eLife. 2020 Oct 7;9:e60225. doi: 10.7554/eLife.60225 (PMC7644213; doi:10.7554/eLife.60225)
Supplement: Transparent reporting form [file elife-60225-transrepform.docx]

***eLife’s* transparent reporting form**

We encourage authors to provide detailed information *within their submission* to facilitate the interpretation and replication of experiments. Authors can upload supporting documentation to indicate the use of appropriate reporting guidelines for health-related research (see [EQUATOR Network](http://www.equator-network.org/%20)), life science research (see the [BioSharing Information Resource](https://biosharing.org/)), or the [ARRIVE guidelines](http://www.plosbiology.org/article/info:doi/10.1371/journal.pbio.1000412) for reporting work involving animal research. Where applicable, authors should refer to any relevant reporting standards documents in this form.

If you have any questions, please consult our Journal Policies and/or contact us: [editorial@elifesciences.org](mailto:editorial@elifesciences.org).

**Sample-size estimation**

- You should state whether an appropriate sample size was computed when the study was being designed
- You should state the statistical method of sample size computation and any required assumptions
- If no explicit power analysis was used, you should describe how you decided what sample (replicate) size (number) to use

Please outline where this information can be found within the submission (e.g., sections or figure legends), or explain why this information doesn’t apply to your submission:

Details on sample sizes are described in the respective figure legends. Details on statistical analyses are described in the material and methods section under section “Statistical analyses”.

**Replicates**

- You should report how often each experiment was performed
- You should include a definition of biological versus technical replication
- The data obtained should be provided and sufficient information should be provided to indicate the number of independent biological and/or technical replicates
- If you encountered any outliers, you should describe how these were handled
- Criteria for exclusion/inclusion of data should be clearly stated
- High-throughput sequence data should be uploaded before submission, with a private link for reviewers provided (these are available from both GEO and ArrayExpress)

Please outline where this information can be found within the submission (e.g., sections or figure legends), or explain why this information doesn’t apply to your submission:

Details on sample sizes are described in the respective figure legends. Biological replicates are defined as independent individuals: mice or embryos/explants.

For organoid experiments, replicates correspond to independent experiments performed on cells sorted from independent mice. For figure 1H and figure1-figure supplement 3C, the total number of organoids quantified is indicated above the chart, and correspond to counts from 2 independent experiments and N=2 mice.

For single-cell RNA-seq experiments, the number of biological replicates for each condition are reported in supplementary file 1.

**Statistical reporting**

- Statistical analysis methods should be described and justified
- Raw data should be presented in figures whenever informative to do so (typically when N per group is less than 10)
- For each experiment, you should identify the statistical tests used, exact values of N, definitions of center, methods of multiple test correction, and dispersion and precision measures (e.g., mean, median, SD, SEM, confidence intervals; and, for the major substantive results, a measure of effect size (e.g., Pearson's r, Cohen's d)
- Report exact p-values wherever possible alongside the summary statistics and 95% confidence intervals. These should be reported for all key questions and not only when the p-value is less than 0.05.

Please outline where this information can be found within the submission (e.g., sections or figure legends), or explain why this information doesn’t apply to your submission:

Statistical analyses were performed using Graphpad/Prism (v8.4.2). Data are represented as mean ±SD. Unless otherwise specified in the corresponding figure legend, two-tailed unpaired *t*-tests were used to compare means between two groups. Statistical significance was set at p < 0.05. For animal model studies, no statistical method was used to pre-determine the sample size. No randomization or blinding was used for *in vivo* studies.

(For large datasets, or papers with a very large number of statistical tests, you may upload a single table file with tests, Ns, etc., with reference to sections in the manuscript.)

**Group allocation**

- Indicate how samples were allocated into experimental groups (in the case of clinical studies, please specify allocation to treatment method); if randomization was used, please also state if restricted randomization was applied
- Indicate if masking was used during group allocation, data collection and/or data analysis

Please outline where this information can be found within the submission (e.g., sections or figure legends), or explain why this information doesn’t apply to your submission:

Mice were allocated in the respective experimental groups based on their genotypes to have ≥ N=3 biological replicates per condition. No statistical method was used to pre-determine the sample size. No randomization or blinding was used for *in vivo* studies.

**Additional data files (“source data”)**

- We encourage you to upload relevant additional data files, such as numerical data that are represented as a graph in a figure, or as a summary table
- Where provided, these should be in the most useful format, and they can be uploaded as “Source data” files linked to a main figure or table
- Include model definition files including the full list of parameters used
- Include code used for data analysis (e.g., R, MatLab)
- Avoid stating that data files are “available upon request”

Please indicate the figures or tables for which source data files have been provided:

Raw sequencing files and processed gene expression matrices have been deposited in the NCBI Gene Expression Omnibus under the accession number GSE151944. The processed datasets for both mouse adult prostate and UGS prostate explant cultures can be accessed via a searchable R Shiny application available at <http://shiny.cruk.manchester.ac.uk/pscapp/>. All code used to process data and generate figures is available on a public GitHub repository at <https://github.com/glacaud/prostate-scRNAseq>.
